# Supplementary material for: A clinical and mechanistic study of topical borneol‐induced analgesia
Source: EMBO Mol Med. 2017 Apr 10;9(6):802–15. doi: 10.15252/emmm.201607300 (PMC5452010; doi:10.15252/emmm.201607300)
Supplement: Supplementary file 1 — Appendix [file EMMM-9-802-s001.pdf]

A clinical and mechanistic study of topical borneol-induced analgesia

Shu Wang, Dan Zhang, Jinsheng Hu, Qi Jia, Wei Xu, Deyuan Su, Hualing Song, Zhichun Xu, Jianmin Cui, Ming Zhou, Jian Yang and Jianru Xiao

Appendix

Appendix Table S1

Appendix Table S1

		P value
**Figure 2**		
2A	Ethanol + Saline VS Ethanol + Cap	1.13E-08
	25% Borneol + Cap VS Ethanol + Cap	4.36E-08
2B	Ethanol + Saline VS Ethanol + Cap	3.91E-12
	25% Borneol + Cap VS Ethanol + Cap	6.98E-09
2C	Phase 1	
	1.5% Borneol VS 0% Borneol	4.52E-03
	4.5% Borneol VS 0% Borneol	1.59E-03
	15% Borneol VS 0% Borneol	1.19E-04
	Phase 2	
	4.5% Borneol VS 0% Borneol	1.59E-03
	15% Borneol VS 0% Borneol	9.26E-05
2D	Ethanol (Ipsilateral) VS borneol (Ipsilateral)	
	1 day	4.11E-05
	2 day	4.77E-03
	3 day	3.93E-03
	7 day	3.00E-02
	Ethanol (Ipsilateral) VS Ethanol (Contralateral)	
	1 day	8.59E-05
	2 day	9.44E-07
	3 day	7.43E-07
	7 day	2.53E-02
2E	Ethanol + CFA VS Ethanol + Saline	
	1 day	2.68E-03
	4 day	7.03E-06
	7day	2.77E-02
	Ethanol + CFA VS Borneol + CFA	
	1 day	1.49E-02
	4 day	1.14E-02
**Figure 3**		
3A	Ethanol + Saline VS Ethanol + Cap	1.33E-08
	15% Borneol + Cap VS Ethanol + Cap	1.41E-05
3B	Ethanol + Saline VS Ethanol + Cap	1.91E-14
	15% Borneol + Cap VS Ethanol + Cap	6.76E-12
3C	Borneol + CFA VS Ethanol + CFA	
	4 day	2.18E-02
	7 day	1.12E-02
	Ethanol + CFA VS Ethanol + Saline	

	1 day	4.47E-02
	4 day	6.54E-04
	7 day	1.61E-02
3D	Saline VS Bicuculline	2.70E-01
3E	Saline VS Bicuculline	9.70E-01
3F	Saline VS Muscimol	4.53E-09
	Muscimol + Bicuculline VS Muscimol	3.19E-04

Figure 5

5B	Phase 1	
	Ethanol (WT) VS Ethanol (TRPM8$^{-/-}$)	9.02E-01
	15% Borneol (WT) VS 15% Borneol (TRPM8$^{-/-}$)	1.66E-02
	Phase 2	
	Ethanol (WT) VS Ethanol (TRPM8$^{-/-}$)	8.25E-01
	15% Borneol (WT) VS 15% Borneol (TRPM8$^{-/-}$)	1.76E-03
5C	Ethanol (Ipsilateral) VS borneol (Ipsilateral)	
	1 day	9.60E-01
	2 day	2.47E-01
	3 day	4.63E-01
	7 day	4.87E-01
	10 day	1.70E-01
	14 day	7.49E-01
5D	Ethanol + CFA VS Borneol+ CFA	
	1 day	6.77E-01
	4 day	5.87E-01
	7 day	5.41E-01
5F	Phase 1	
	Ethanol(Saline) VS Ethanol(AMTB)	8.95E-01
	Borneol(Saline) VS Borneol(AMTB)	1.45E-02
	Phase 2	
	Ethanol(Saline) VS Ethanol(AMTB)	9.40E-01
	Borneol(Saline) VS Borneol(AMTB)	5.48E-04

Figure 6

6A	Ethanol VS 15% Borneol	2.44E-07
	Ethanol VS 15% Menthol	2.43E-07
	15% Borneol VS 15% Menthol	7.51E-01
6B	Phase 1	
	Ethanol VS 15% Borneol	7.15E-03
	Ethanol VS 15% Menthol	4.61E-03
	15% Borneol VS 15% Menthol	4.12E-01

	Phase 2	
	Ethanol VS 15% Borneol	1.55E-03
	Ethanol VS 15% Menthol	4.11E-03
	15% Borneol VS 15% Menthol	7.31E-01
6C	Ethanol + Saline VS Ethanol + Cap	2.80E-10
	Ethanol + Cap VS 15% Bornoel + Cap	9.30E-02
	Ethanol + Cap VS 15% Menthol + Cap	6.33E-05
6D	Ethanol + CFA VS Ethanol + Saline	
	1 day	1.43E-02
	4 day	7.50E-03
	7 day	1.60E-02
	Ethanol + CFA VS Menthol + CFA	
	1 day	1.73E-02
	4 day	4.82E-02
6E	Saline VS Naloxone	5.27E-01
6F	Saline VS Naloxone	2.13E-03
6G	Saline VS Naloxone	5.56E-01
6H	Saline VS Naloxone	7.89E-01
6I	Saline VS LY341495	9.05E-01
6J	Saline VS LY341495	4.49E-03
6K	Ethanol VS 15% Borneol	2.99E-01
	Ethanol VS 15% Menthol	4.17E-04
6L	Ethanol VS 15% Borneol	5.98E-01
	Ethanol VS 15% Menthol	2.34E-05

**Fig. EV4**	A	Saline VS naloxone	4.70E-03
	B	Morphine VS Morphine + Naloxone	1.82E-06

**Fig. EV5**	Ethanol VS 15% Menthol	5.40E-10
