# Supplementary material for: A clinical and mechanistic study of topical borneol‐induced analgesia
Source: EMBO Mol Med. 2017 Apr 10;9(6):802–15. doi: 10.15252/emmm.201607300 (PMC5452010; doi:10.15252/emmm.201607300)
Supplement: Supplementary file 2 — Expanded View Figures PDF [file EMMM-9-802-s002.pdf]

Expanded View Figures

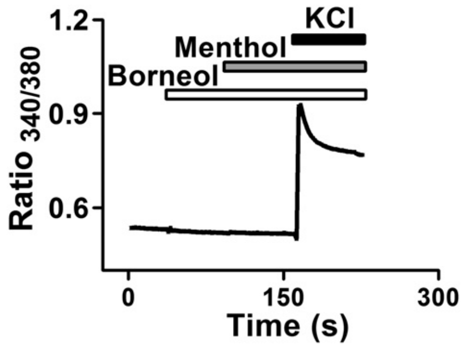

Figure EV1. Borneol has no effect on DRG neurons from TRPM8$^{-/-}$ mice.
Averaged intracellular Ca^{2+} increases in TRPM8$^{-/-}$ DRG neurons in response to consecutive applications of 200 µM borneol, 200 µM menthol, and 67 mM KCl. A total of 1,127 KCl-responsive neurons from four mice were included in the analysis, and none of the neurons responded to borneol.

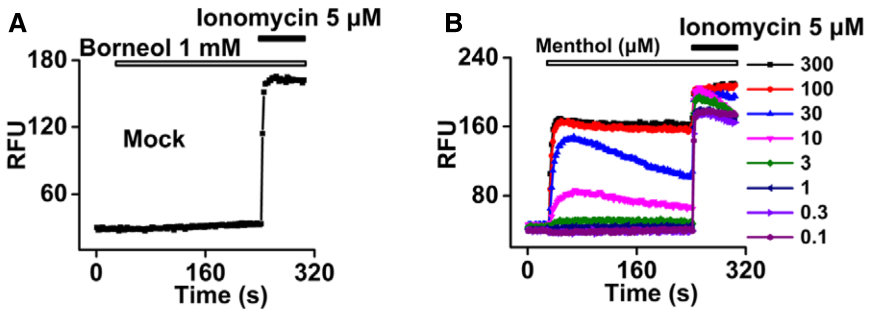

Figure EV2. The borneol effect on mock-transfected HEK 293 cells and the menthol effect on TRPM8-expressing cells.

A Representative intracellular Ca^{2+} signals in HEK 293 cells transfected with empty vector in response to borneol and subsequent applied Ca^{2+} ionophore ionomycin (n = 6).

B Representative intracellular Ca^{2+} signals in HEK 293 cells expressing hTRPM8 in response to different concentrations of menthol and the subsequently applied Ca^{2+} ionophore ionomycin.

© 2017 The Authors

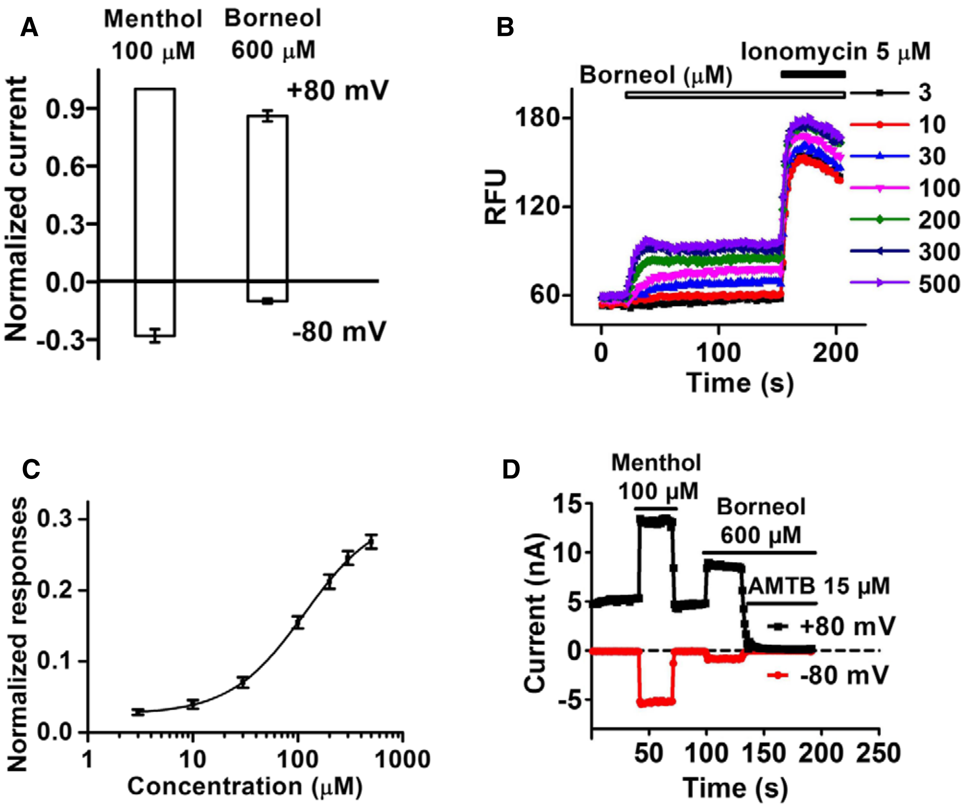

Figure EV3. Borneol activates TRPM8.

A Quantification of consecutively applied 100 μM menthol- and 600 μM borneol-induced hTRPM8 currents. Currents were normalized to 100 μM menthol-induced currents at +80 mV (n = 6).

B Representative intracellular Ca^{2+} signals in HEK 293 cells expressing mouse TRPM8 in response to different concentrations of borneol.

C Dose–response curves of borneol-induced increase in intracellular Ca^{2+} in mouse TRPM8-expressing HEK 293 cells. The smooth curve is a fit to the Hill equation with an EC_{50} of 116 μM (n = 12). The data were normalized to ionomycin-induced intracellular Ca^{2+} increases.

D Time course of menthol- and subsequently applied borneol-induced whole-cell currents in mouse TRPM8-expressing HEK 293 cells (n = 5).

Data information: All the data are presented as the mean ± standard error of the mean (SEM).

 © 2017 The Authors

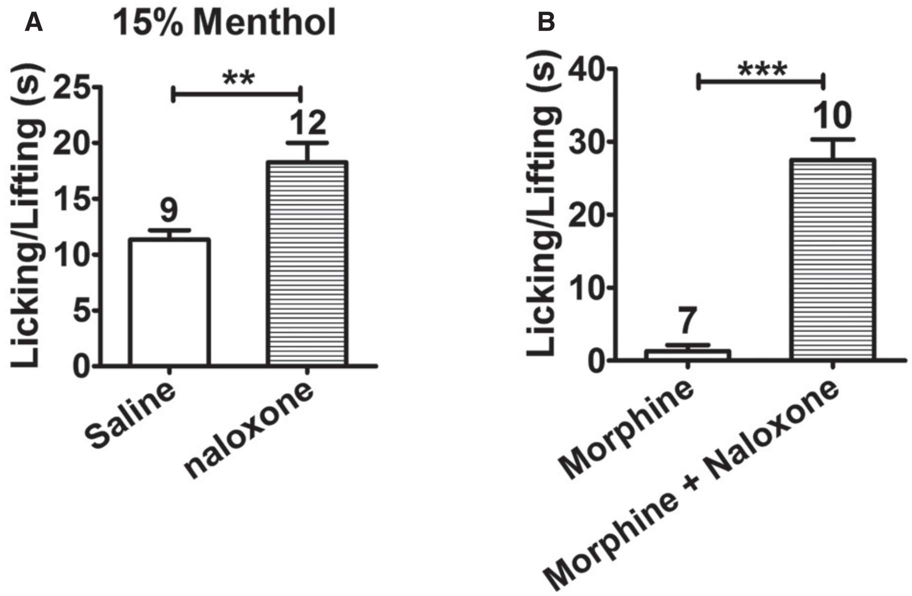

Figure EV4. Intrathecal naloxone antagonizes menthol- or morphine-induced analgesia.

A Quantification of the effect of intrathecal injection of naloxone on menthol-induced analgesia in $TRPM8^{-/-}$ mice. After control saline or naloxone was intrathecally injected in $TRPM8^{-/-}$ mice, 15% menthol was applied to a hindpaw for a total of three times. After 10 min, 100 μM Cap was injected into the paw, and paw licking and lifting time was measured within 5 min.

B Quantification of 100 μM Cap-induced nociceptive responses in WT mice after intrathecal injection of morphine with or without naloxone.

Data information: The number of mice is indicated on top of each bar. Statistical significance was evaluated using two-tailed *t*-test. $^{**}P < 0.01$; $^{***}P < 0.001$; the exact *P*-values are indicated in Appendix Table S1. All the data are presented as the mean ± standard error of the mean (SEM).

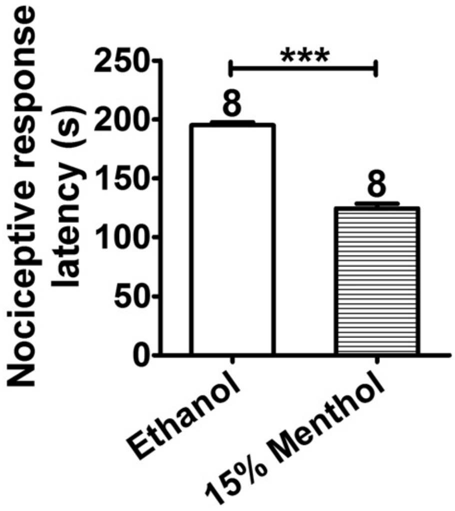

Figure EV5. Menthol causes cold hypersensitivity in $TRPM8^{-/-}$ mice.
Ethanol or menthol was applied to both hindpaws of $TRPM8^{-/-}$ mice, and the nociceptive response latencies were measured in a cold plate test (0°C). Statistical significance was evaluated using two-tailed *t*-test. $^{***}P < 0.001$; the exact *P*-value is indicated in Appendix Table S1. The number of mice is indicated on top of each bar. All the data are presented as the mean ± standard error of the mean (SEM).

© 2017 The Authors
